# Assembly of a Comprehensive Regulatory Network for the Mammalian Circadian Clock: A Bioinformatics Approach

**DOI:** 10.1371/journal.pone.0126283

**Published:** 2015-05-06

**Authors:** Robert Lehmann, Liam Childs, Philippe Thomas, Monica Abreu, Luise Fuhr, Hanspeter Herzel, Ulf Leser, Angela Relógio

**Affiliations:** 1 Institute for Theoretical Biology (ITB), Charité-Universitätsmedizin Berlin and Humboldt-Universität zu Berlin, Invalidenstraße 43, 10115, Berlin, Germany; 2 Knowledge Management in Bioinformatics, Institute for Computer Science, Humboldt-Universität zu Berlin, Unter den Linden 6, 10099, Berlin, Germany; 3 Molekulares Krebsforschungszentrum (MKFZ), Charité-Universitätsmedizin Berlin, Augustenburger Platz 1, 13353, Berlin, Germany; McGill University, CANADA

## Abstract

By regulating the timing of cellular processes, the circadian clock provides a way to adapt physiology and behaviour to the geophysical time. In mammals, a light-entrainable master clock located in the suprachiasmatic nucleus (SCN) controls peripheral clocks that are present in virtually every body cell. Defective circadian timing is associated with several pathologies such as cancer and metabolic and sleep disorders. To better understand the circadian regulation of cellular processes, we developed a bioinformatics pipeline encompassing the analysis of high-throughput data sets and the exploitation of published knowledge by text-mining. We identified 118 novel potential clock-regulated genes and integrated them into an existing high-quality circadian network, generating the to-date most comprehensive network of circadian regulated genes (NCRG). To validate particular elements in our network, we assessed publicly available ChIP-seq data for BMAL1, REV-ERBα/β and RORα/γ proteins and found strong evidence for circadian regulation of *Elavl1*, *Nme1*, *Dhx6*, *Med1* and *Rbbp7* all of which are involved in the regulation of tumourigenesis. Furthermore, we identified *Ncl* and *Ddx6*, as targets of RORγ and REV-ERBα, β, respectively. Most interestingly, these genes were also reported to be involved in miRNA regulation; in particular, NCL regulates several miRNAs, all involved in cancer aggressiveness. Thus, NCL represents a novel potential link via which the circadian clock, and specifically RORγ, regulates the expression of miRNAs, with particular consequences in breast cancer progression. Our findings bring us one step forward towards a mechanistic understanding of mammalian circadian regulation, and provide further evidence of the influence of circadian deregulation in cancer.

## Introduction

Almost all organisms evolved an endogenous circadian clock which regulates the timing of central biological processes and provides a way to adapt physiology and behaviour to daily dark/light rhythms [1–3]. In mammals, malfunctions of the circadian system are associated to known pathologies ranging from sleep or metabolic disorders, to cancer [4–6]. Hence, a detailed overview of the underlying genetic network that shapes the mammalian circadian system is of major interest to the circadian and medical field.

The mammalian circadian system is hierarchically organized. A main pacemaker formed by two clusters of ~100,000 neurons (in humans) is located in the suprachiasmatic nucleus (SCN), but peripheral oscillators exist in virtually every of our 3.5×10^13^ body cells [7, 8]. Extensive research has identified a reduced set of 14 genes to form the so called core-clock network (CCN), within a cell. These genes encode for members of several gene families: PER (period), CRY (cryptochrome), BMAL (brain and muscle ARNT-like protein), CLOCK (circadian locomotor output cycles kaput), NPAS2 (neuronal PAS domain-containing protein 2, in neuronal tissue), ROR (retinoic acid receptor-related orphan receptor) and REV-ERB (nuclear receptor, reverse strand of ERBA). The CCN is arranged in two main interconnected feed-back loops: a) the RORs/*Bmal*/REV-ERBs (RBR) loop and b) the PERs/CRYs (PC) loop [9]. Both loops are able to produce rhythms in gene expression, independently, but need to be interconnected to robustly generate oscillations with a period of circa 24 hours [10, 11]. In the centre of the core-clock network lays the heterodimer complex CLOCK/BMAL1. This complex regulates the transcription of elements of both the RBR and PC loop by binding to E-Box sequences in the promoter region of the target genes. In the RBR-loop, *Rev-Erbα*,*β* and *Rorα*,*β*,*γ* are transcribed. After translation, the resulting proteins compete for RORE elements within the *Bmal1* promoter region and hold antagonistic effects, thereby fine-tuning *Bmal1* expression. In the PC loop, following transcription and translation, PER1,2,3 and CRY1,2 form complexes and inhibit CLOCK/BMAL mediated-transcription, thus regulating the expression of all target genes mentioned above.

The CCN has been studied, on a fine scale, at the transcriptional, translational and post-translational level both experimentally and with mathematical models [9, 12–18]. Furthermore, various efforts have been made to decipher the mechanisms through which the mammalian CCN regulates its target genes, the clock-controlled genes (CCG), as well as to identify new CCGs [19–21]. Yet, a more detailed knowledge on the full range of genes and subsequent biological processes that are regulated by the core of the circadian clock is still missing. Therefore, a comprehensive analysis of the relevance of such connections, as well as on the putative effects of deregulations on circadian output and resulting pathological phenotypes, is needed.

**Fig 1:**
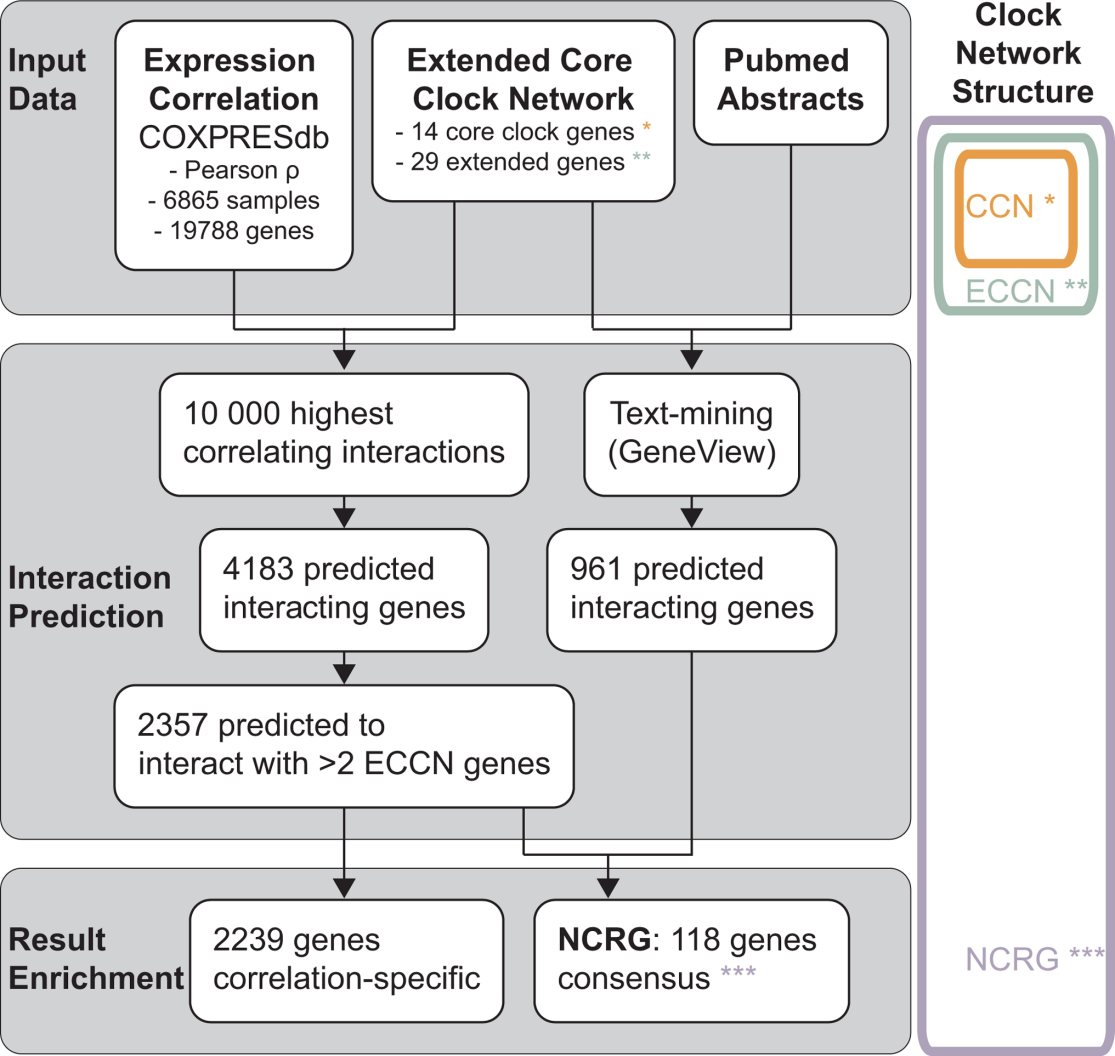
Work flow used to establish a network of circadian regulated genes (NCRG). Two independent data types were used to predict genes which interact with the human core clock network (CCN, orange) and the extended core clock network (ECCN, green). Co-expression data was used to find sets of genes with strongest (anti-) correlating expression with the 43 ECCN genes across a large number of independent experiments. A total of 2357 genes were found to interact with more than 2 ECCN genes. The GeneView text-mining pipeline was used to analyse published knowledge (approximately 22 million citations) about interacting genes. A total of 961 text-mining-predicted genes were found. The intersection of both methodologies resulted in 118 new genes, which together with the ECCN form a new network of circadian regulated genes (NCRG, purple).

In this manuscript, we present a comprehensive mammalian circadian network constructed by an integrated bioinformatics pipeline which uses different data sources and different data types. This novel circadian network topology highlights particularly genes which link the circadian clock to several biological processes, often in multiple alternative ways. We carried out a systematic expansion of a previously published core-clock network (ECCN) using gene co-expression analysis, text-mining on the full PubMed, signatures of circadian expression patterns, and ChIP-Seq data. We used the first two of these methods to identify a set of 118 novel high-confidence ECCN target genes, whereas the latter two data types were used for validation of this set, which resulted in a novel network of circadian regulated genes (NCRG) (Fig 1). In particular, ChIP-seq data for BMAL1, RORα,γ and REV-ERBα,β [12, 15–17, 22] confirmed links between the ECCN and several cancer-related genes. Notably, two of these genes were shown to be involved in miRNA regulation.

Altogether, our findings suggest, new potential clock genes and describe their role and topology within the circadian network. Our work delivers novel evidence to the influence of circadian deregulation in cancer and adds a novel way via which a clock-dependent cancer output may emerge, i.e., miRNA circadian regulation.

## Results

### A text-mining based approach for network discovery

We aimed to update and extend our recently reported circadian network (ECCN-extended core-clock network) [21]. For that we combined the results of a text-mining system with high-throughput gene co-expression data to obtain new elements and interactions. This procedure resulted in a network of circadian regulated genes (NCRG) following the workflow schematized in Fig 1.

**Fig 2:**
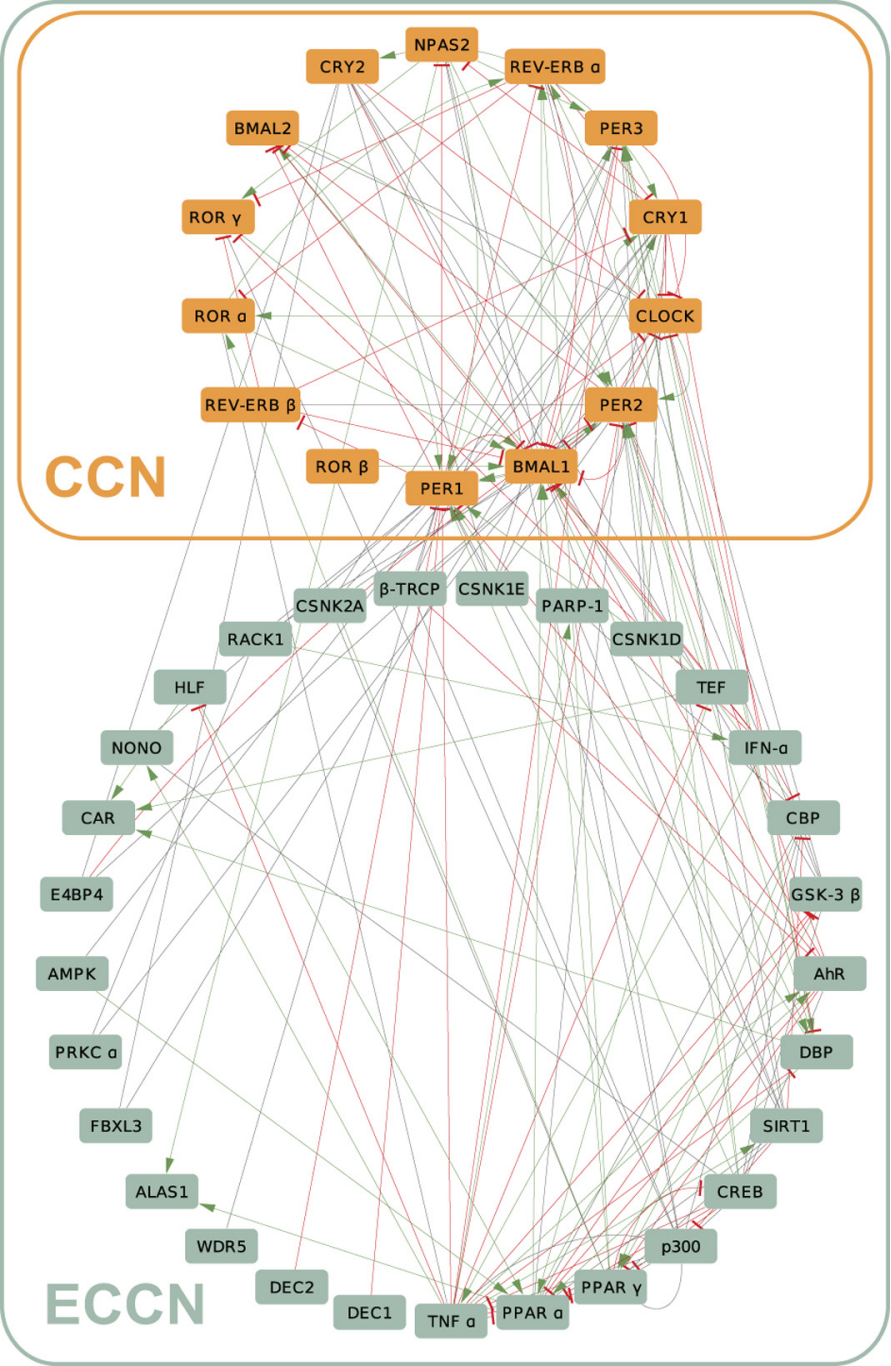
The human core clock network (CCN) and the extended core clock network (ECCN). The CCN (orange) contains the known core-clock elements (Per1,2,3, Cry1,2, Rev-Erbα,β, Rorα,β,γ, Bmal1,2, Clock and Npas). The ECCN (green) was obtained after an extensive collection of CCN-interacting genes followed by a detailed curation for direct interactions [21] and a further update to the recent literature. Activation (green lines), inhibition (red lines) and other sort of interactions (grey lines) are represented. The resulting clock network contains 43 elements and more than 200 regulatory relationships.

The original ECCN [21] contains a core of 14 well known circadian genes, *Per1*,*2*,*3*, *Cry1*,*2*, *Bmal1*,*2*, *Rorα*,*β*,*γ and Rev-Erbα*,*β* as well as *Clock*, its paralog *Npas2*, and their direct neighbouring targets. We started our study by generating an update version of the ECCN using the text-mining software—GeneView (see Materials and Methods) [23] to extract all pairwise interactions among our genes of interest and their directly interacting neighbours. The new ECCN contains 43 elements as the previous network [21], the depicted interactions were updated to the current PubMed available data resulting in more than 200 regulatory relationships (Fig 2). Additional information containing all interactions and corresponding references, as well as a more detailed characterization of the ECCN is provided in S1 Table and S1 Text, respectively.

### Co-expression data analysis confirms the ECCN network topology

In this work we expanded the updated ECCN with a new layer of potentially ECCN-regulated elements (genes and proteins) using co-expression data as a first source of evidence. We consider as such candidates all genes which show a strong co-expression to ECCN members and which can be confirmed using text-mining, as indicated in Fig 1.

**Fig 3:**
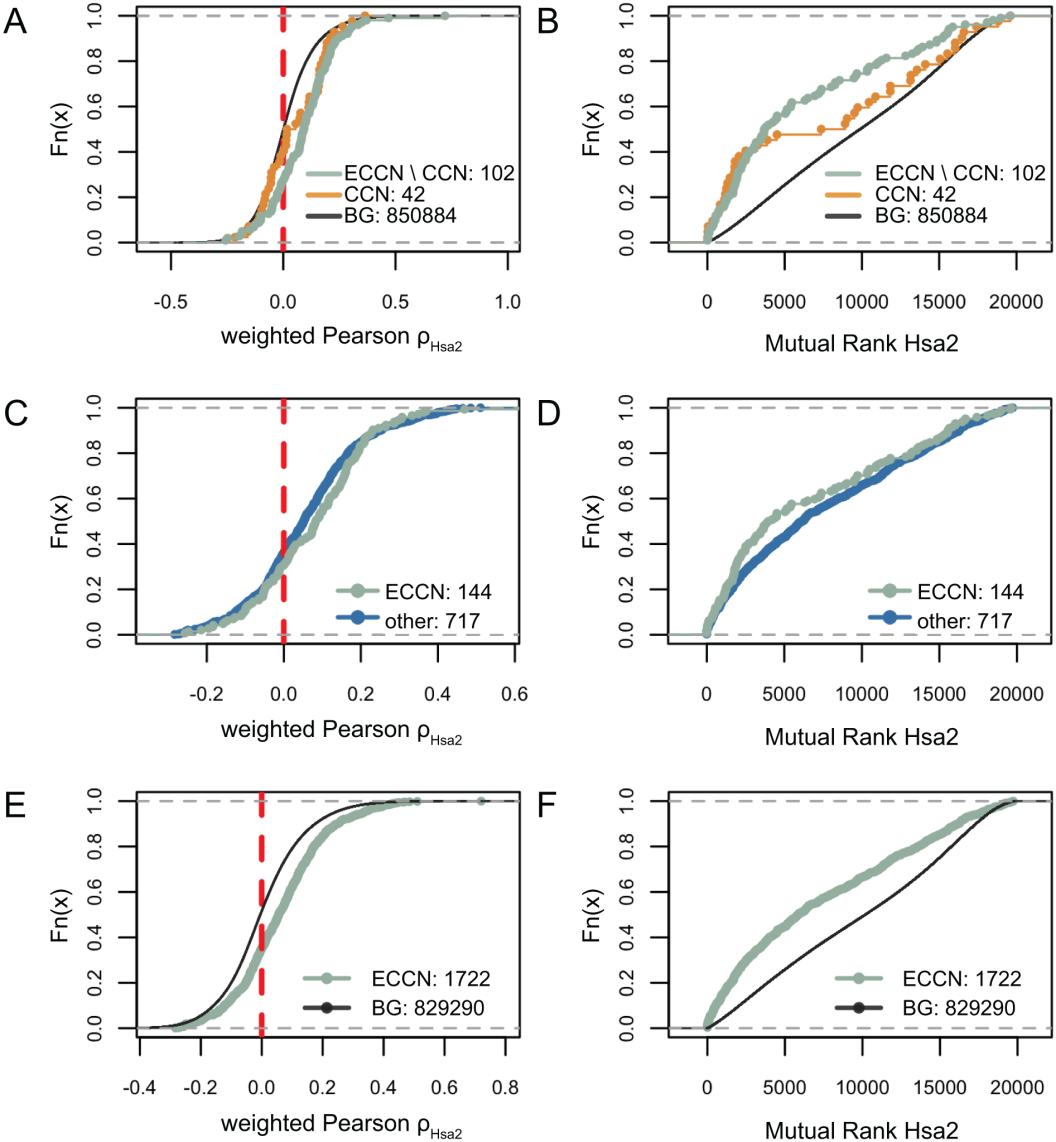
Correlation distributions for clock network gene pairs versus random gene pairs. The cumulative Pearson ρ distributions of pairs of ECCN genes reported to interact but excluding CCN (ECCN, green), reported pairs of CCN genes (CCN, orange), and 43 randomly chosen genes versus all genes as background (BG, black) are shown for the Hsa2 data collection **(A, B)**. Distributions are shown centred around 0 with the centred bin marked by the dashed red line. The Pearson ρ distributions of reported pairs of ECCN genes (green) is compared to not reported pairs (blue) for the data set Hsa2 **(C, D).** Comparison of Pearson ρ and mutual rank **(E, F)** between all possible pairs of ECCN genes (green) and all possible pairs of non-ECCN genes as background (black). All data were taken from the Hsa2 data collection.

There are several public available databases providing co-expression metrics for human genes, we evaluated four different such databases [24–26] regarding their ability to reproduce the ECCN, to find the best suited for our analysis. Results of our comparisons are presented in S2 Text. We eventually choose COXPRESdb [25] (see Fig 3), as this database showed the highest degree of correlation within all genes of the extended core-clock network.

We expected a significant difference between the correlation measure distributions of interacting gene pairs and a chosen background, if unknown interacting pairs were to be predicted based on correlation values. All possible pairs between a random set of 43 genes and all genes (19,788) were used as background. As foreground pairs we used the 42 known available interactions amongst the CCN genes, as well as the 119 curated interactions of the ECCN gene set. Both the CCN (orange) and ECCN (green) gene pairs tended to have higher correlations compared to the random background and thus lower mutual rank (MR) values (Fig 3A and 3B and S1 Fig). The probability density functions of correlation and mutual rank are shown in S2 Fig for both datasets. All correlation values were Fisher transformed to ensure normal distribution prior to hypothesis testing to characterize differences between the CCN, ECCN, and the background. Subsequent one-sided t-tests with the alternative hypothesis to observe smaller correlations in the background confirmed the results of the visual inspection: CCN gene pairs are significantly higher correlated than the background pairs (p < 0.0195) similar to the ECCN gene pairs (p < 6e^-7^). Similarly significant differences were observed in the Hsa dataset for the CCN (p < 1.4e^-3^) and the ECCN (p < 1.2e^-7^). Furthermore, no significant difference was found between correlations in the CCN and the ECCN for the Hsa (p < 0.41) and the Hsa2 dataset (p < 0.18).

Studying the co-expression of ECCN genes in detail, we observed anti-correlated circadian expression profiles between *Per* and *Bmal*, which is consistent with the predicted 9h delay between the mRNAs peak expression for these genes [9]. The strongest anti-correlation was determined between ρ_HSA2_(*Bmal1*,*Per3*) = -0.21 and the weakest between ρ_HSA2_(*Bmal2*,*Per2*) = -0.054. For the expected expression-correlation between *Ror* and *Rev-Erb* (circa 7h delay), we determined weaker anti-correlations, with ρ_HSA2_(*Rev-Erbβ*,*Rorγ*) = -0.04, ρ_HSA2_(*Rev-Erbα*,*Rorγ*) = 0.06, and ρ_HSA2_(Rev-Erbα,Rorα) = 0.16. We could also validate the expected positive correlation between *Per* and *Cry*, specifically for *Per2/Cry2* with ρ_HSA2_(*Per2*,*Cry2*) = 0.31. For other *Per* and *Cry* family members, smaller correlation values were found with ρ_HSA2_(*Per2*,*Cry1*) = 0.12, and ρ_HSA2_(*Per1*,*Cry1*) = 0.0022.

Next, we tested whether the distribution of correlations between pairs of reported interacting ECCN genes could be distinguished from all other possible pairs of ECCN genes. The known interactions exhibited positive ρ_HSA2_ values and thus lower MR (Fig 3C and 3D). However, only a weak tendency of known interactions towards higher correlations compared to non-reported pairs can be observed (probability density functions shown in S3 Fig). The corresponding comparisons of correlation distributions via t-tests confirmed the weak tendency in the Hsa2 dataset (p < 0.059) whereas no signal was found in the Hsa dataset (p < 0.395). As a consequence, we conclude that known interacting gene pairs cannot reliably be distinguished from other pairs within the ECCN based merely on expression correlation. We then tested the assumption that expression patterns are generally higher correlated within the ECCN as compared to other non-ECCN gene pairs. The resulting distributions for Pearson ρ and mutual rank between all possible pairs of the ECCN genes included in the datasets, as compared to all combinations of the same genes with all other genes are shown in Fig 3E and 3F (probability density functions shown in S4 Fig). We confirmed the difference of the ECCN set as compared to the background set as before with t-test which yielded high significance in both datasets (p < 2.2e^-16^). In addition, the corresponding mutual rank measures were also found to be significantly lower (Wilcoxon Rank Sum test, p < 2.2e^-16^). Hence, we concluded that the examined expression correlation data provided information about the membership of a gene to the clock network, but not about the network’s topology.

### Expression correlation-based target prediction

**Fig 4:**
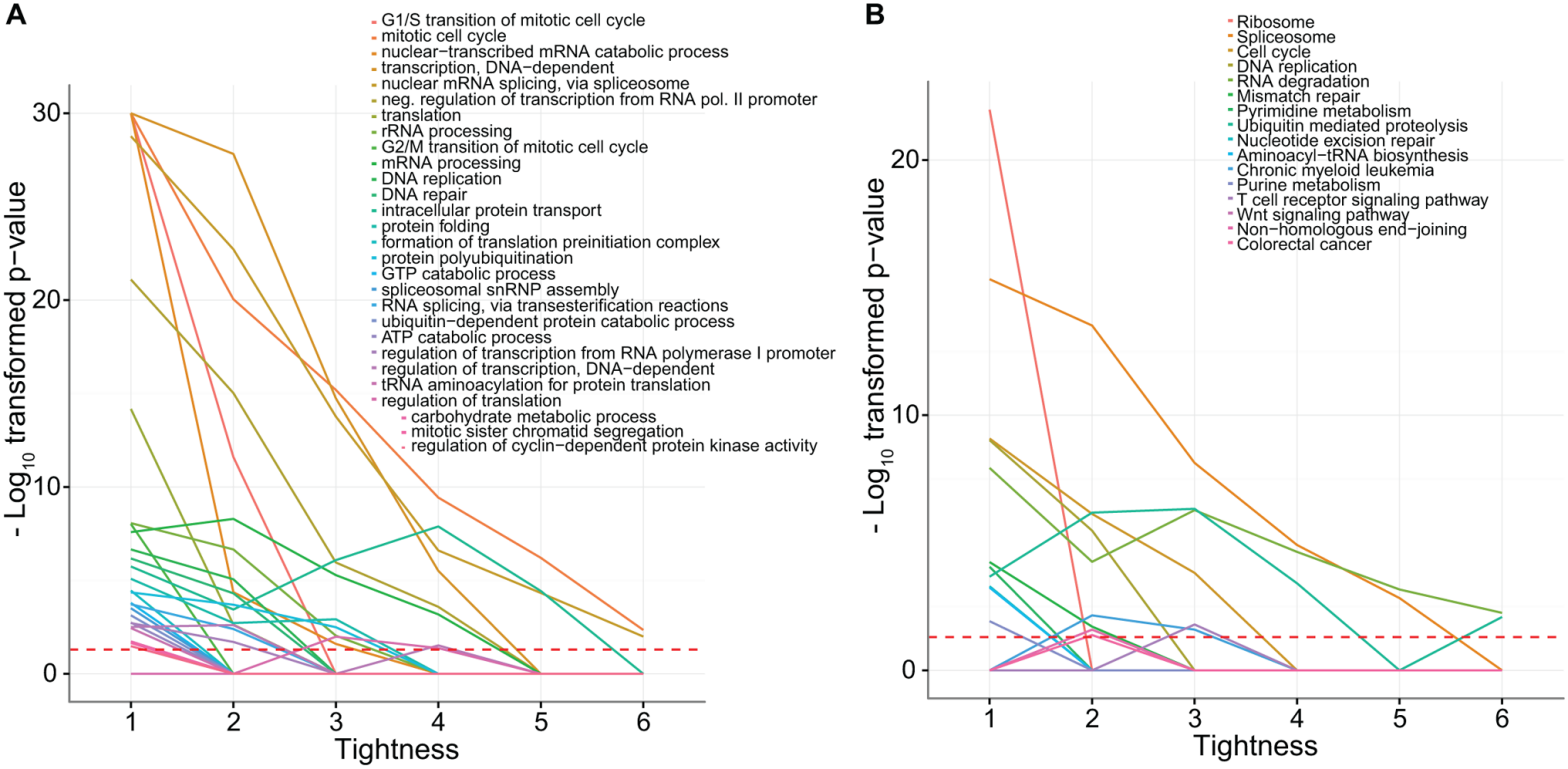
Variation in functional annotation enrichment with increasing tightness between the predicted targets and the ECCN. The significance of 28 enriched GO terms **(A)** and 16 KEGG terms **(B)** for genes connected to the CCN steadily decreases for most terms as the minimum number connections is increased (this number of connections between a gene and a gene set is here defined as "tightness"). As the minimum tightness between the predicted targets and the ECCN increases, the enrichment and rank of functional annotation changes. We observe an overall decrease in enrichment but little in rank. The greatest changes in rank occur between a tightness of 2 and 3. At a tightness of two and above, the rank of the majority of significant GO terms such as "mitotic cell cycle" and "nuclear mRNA splicing, via spliceosome", and KEGG terms such as "Spliceosome", "Ubiquitin mediated proteolysis" and "RNA degradation" remain largely stable suggesting a natural threshold on tightness at this point.

We selected the 10.000 highest correlating pairs of one of the 43 ECCN genes and any other gene. This conservatively chosen threshold selects 1.18% of all pairs, corresponding to an absolute correlation cut-off of 0.3636, or 2.6 σ. In this set, the number of unique new genes was 4.183. As we sought to investigate genes that were tightly associated with the ECCN (i.e. associated with multiple ECCN genes). We defined tightness as the number of connections between a gene and the ECCN and sought to find the largest number of tightly connected predicted targets by filtering them at several levels of minimum tightness. At increasing levels of minimum tightness, we performed an overrepresentation analysis of GO and KEGG terms for varying minimal values of these counts. The largest changes in overrepresented terms occurred when changing the threshold from one to two (Fig 4). There, terms related to cell cycle and the ribosome rapidly dropped in significance, while, terms related to splicing and transcription largely retained their position. When increasing the tightness further, much smaller changes occurred.

Accordingly, we defined tightly connected genes as those having two or more associations with the ECCN, which reduced the number of predicted ECCN targets from 4,183 to 2,357 with 8,180 interactions (S5 Fig).

The number of tightly connected genes associated to a given ECCN element varied greatly. While 11 ECCN members did not feature any interaction, the three elements CREB, AMPK, and CLOCK covered 48% of all predicted interactions (S6 Fig).

**Fig 5:**
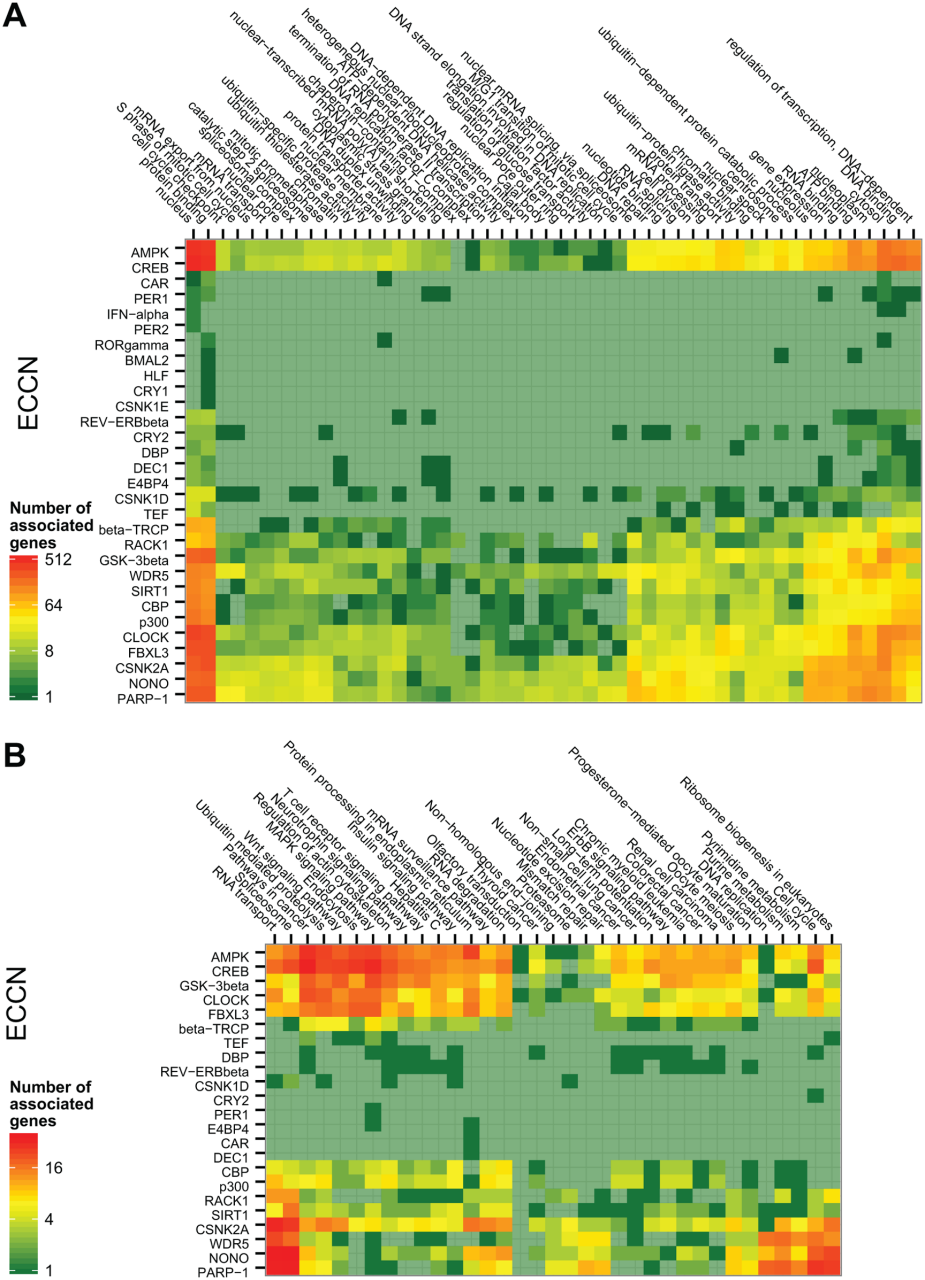
Homogenous functional spectrum of genes targeted by different ECCN genes. The specific functions of genes interacting with each individual ECCN gene were counted and illustrated as heat map. Annotated KEGG pathways **(A)** and GO terms **(B)** found to be overrepresented amongst all predicted target genes in the preceding analysis were counted for each target gene, and these counts were then accumulated for each individual ECCN gene and represented as colours according to the legend. Rows and columns are ordered according to a hierarchical clustering.

Gene ontology terms (GO) and KEGG pathway enriched in this set are listed in Table 1 and Table 2, respectively. To obtain an insight into which ECCN genes are of particular importance for the enriched function or pathway, we determined the cross table for each combination of ECCN gene versus enriched term, counting the number of predicted target genes featuring the corresponding term. This approach yielded a consistent pattern for GO functional annotations (50 terms with q < 0.01) and KEGG pathways (35 pathways with q < 0.01) (Fig 5). About half of the ECCN genes were associated with a multitude of genes covering a range of GO annotations, while the other half was associated with few genes covering only a small number of GO terms (Fig 5A). The largest number of target genes was annotated with the molecular function “protein binding” and the cellular component “nucleus”. The second-strongest molecular function signal was “DNA binding”. The most striking association was found between the genes *Csnk2a*, *Wdr5*, *Nono*, and *Parp-1* and the spliceosome (q < 1.5e^-37^) and RNA transport (q < 8e^-38^) pathways, where q represent the p-value adjusted by Benjamini-Hochberg multiple testing correction. These genes were also predicted to target ribosome biogenesis, cell cycle, and purine/pyrimidine synthesis related genes. Another strong association was found between cancer-related pathways such as “Pathways in cancer” (q < 7e^-7^),”Wnt signalling” (q < 2.8e^-8^), “MAPK signalling” (q < 4e^-6^), and the *Ampk* and *Creb* target genes (Fig 5B).

**Table 1:** Enrichment analysis of the co-expression-predicted ECCN interacting genes for GO term annotations.

GO Term	Annotations in Total	Annotations in Predicted Set	Expected	FDR
**nuclear mRNA splicing, via spliceosome**	200	88	27	1.9e-10
**cell division**	443	114	59.81	1.6e-09
**mRNA transport**	105	54	14.18	6.7e-08
**DNA strand elongation involved in DNA replication**	34	21	4.59	1.2e-06
**ubiquitin-dependent protein catabolic process**	347	104	46.85	1.7e-06
**S phase of mitotic cell cycle**	130	44	17.55	1.8e-06
**regulation of glucose transport**	69	24	9.32	1.9e-06
**mitotic prometaphase**	84	35	11.34	1.9e-06
**M/G1 transition of mitotic cell cycle**	76	32	10.26	8.4e-06
**mRNA processing**	378	152	51.03	1.9e-05
**gene expression**	4347	874	586.86	0.00027
**cell cycle checkpoint**	234	70	31.59	0.00031
**DNA duplex unwinding**	28	18	3.78	0.00033
**nuclear-transcribed mRNA poly(A) tail shortening**	26	16	3.51	0.00038
**mRNA export from nucleus**	59	28	7.97	0.00042
**protein transport**	1154	222	155.79	0.00047
**DNA-dependent DNA replication initiation**	28	16	3.78	0.00079
**DNA repair**	369	109	49.82	0.00120
**termination of RNA polymerase II transcription**	44	20	5.94	0.00285
**regulation of transcription, DNA-templated**	2735	506	369.23	0.00518
**RNA splicing**	308	121	41.58	0.00989
**protein binding**	6831	1116	889.85	2.6e-29
**RNA binding**	792	248	103.17	7.0e-24
**DNA binding**	2240	454	291.8	5.8e-14
**ATP binding**	1439	292	187.45	9.4e-13
**nucleotide binding**	2294	448	298.83	3.3e-07
**ubiquitin thiolesterase activity**	64	27	8.34	2.6e-05
**chromatin binding**	235	59	30.61	0.00026
**ubiquitin-protein ligase activity**	241	63	31.39	0.00104
**translation initiation factor activity**	50	21	6.51	0.00134
**ubiquitin-specific protease activity**	43	19	5.6	0.00186
**ATP-dependent DNA helicase activity**	32	17	4.17	0.00547
**protein transporter activity**	87	28	11.33	0.00865
**nucleus**	5640	1172	724.54	2.1e-30
**nucleoplasm**	1401	423	179.98	1.0e-27
**nuclear speck**	144	61	18.5	1.3e-15
**nucleolus**	589	154	75.67	2.4e-14
**catalytic step 2 spliceosome**	78	40	10.02	3.7e-13
**nuclear pore**	60	36	7.71	5.6e-10
**cytosol**	2217	382	284.8	3.6e-07
**heterogeneous nuclear ribonucleoprotein complex**	19	14	2.44	2.5e-06
**centrosome**	363	89	46.63	3.9e-05
**Cajal body**	44	20	5.65	0.00014
**cytoplasmic stress granule**	21	12	2.7	0.00239
**spliceosomal complex**	137	60	17.6	0.00322
**nuclear pore outer ring**	10	8	1.28	0.00330
**chromatin**	280	72	35.97	0.00466
**DNA replication factor C complex**	6	6	0.77	0.00568
**chaperonin-containing T-complex**	6	6	0.77	0.00568
**nuclear membrane**	169	46	21.71	0.00685

Gene ontology annotation enrichment was performed for the molecular function, cellular component, and biological process ontologies. Only terms with q < 0.01 (false discovery rate after Benjamini-Hochberg) are shown.

**Table 2:** Enrichment of KEGG pathway annotations amongst the co-expression-predicted ECCN interacting genes.

KEGG ID	Pathway	p-value	FDR
**hsa03013**	RNA transport	3.51E-40	8.00E-38
**hsa03040**	Spliceosome	6.64E-40	1.50E-37
**hsa04110**	Cell cycle	7.95E-23	1.80E-20
**hsa03008**	Ribosome biogenesis in eukaryotes	6.98E-21	1.60E-18
**hsa04120**	Ubiquitin mediated proteolysis	4.01E-16	9.00E-14
**hsa03018**	RNA degradation	1.95E-14	4.40E-12
**hsa03030**	DNA replication	6.08E-14	1.40E-11
**hsa03015**	mRNA surveillance pathway	8.00E-12	1.80E-09
**hsa04141**	Protein processing in endoplasmic reticulum	2.52E-11	5.60E-09
**hsa04310**	Wnt signalling pathway	1.26E-10	2.80E-08
**hsa05200**	Pathways in cancer	3.19E-09	7.00E-07
**hsa04740**	Olfactory transduction	6.69E-09	1.50E-06
**hsa03430**	Mismatch repair	8.92E-09	1.90E-06
**hsa00230**	Purine metabolism	1.24E-08	2.70E-06
**hsa00240**	Pyrimidine metabolism	1.24E-08	2.70E-06
**hsa04010**	MAPK signalling pathway	1.84E-08	3.90E-06
**hsa03420**	Nucleotide excision repair	6.15E-08	1.30E-05
**hsa05220**	Chronic myeloid leukemia	2.60E-07	5.50E-05
**hsa04722**	Neurotrophin signalling pathway	2.94E-07	6.20E-05
**hsa04144**	Endocytosis	5.12E-07	1.10E-04
**hsa04114**	Oocyte meiosis	7.00E-07	1.50E-04
**hsa05210**	Colorectal cancer	7.63E-07	1.60E-04
**hsa04660**	T cell receptor signalling pathway	1.16E-06	2.40E-04
**hsa05213**	Endometrial cancer	2.74E-06	5.70E-04
**hsa04914**	Progesterone-mediated oocyte maturation	3.32E-06	6.80E-04
**hsa04720**	Long-term potentiation	3.78E-06	7.70E-04
**hsa05160**	Hepatitis C	1.04E-05	2.10E-03
**hsa04810**	Regulation of actin cytoskeleton	1.17E-05	2.40E-03
**hsa04012**	ErbB signalling pathway	1.38E-05	2.80E-03
**hsa05216**	Thyroid cancer	1.42E-05	2.80E-03
**hsa04910**	Insulin signalling pathway	1.45E-05	2.90E-03
**hsa05211**	Renal cell carcinoma	1.76E-05	3.50E-03
**hsa03050**	Proteasome	1.95E-05	3.80E-03
**hsa05223**	Non-small cell lung cancer	2.30E-05	4.50E-03
**hsa03450**	Non-homologous end-joining	5.12E-05	1.00E-02

Only terms with q < 0.01 (false discovery rate after Benjamini-Hochberg) are shown.

### An extended network of circadian regulation: beyond the core

We used text-mining to obtain a second set of genes potentially regulated by the ECCN, and then compared this set to the 2357 genes obtained from co-expression analysis (Fig 1). First, we obtained from GeneView the 50 most frequent interaction partners for each ECCN element, resulting in 961 new interacting genes, each supported by 55 sentences on average. These genes and their supporting sentences are given in S2 Table. The analysis of a large set of GeneView-output sentences revealed 20% of wrong sentences which corresponded to 10% false-positive interactions. Again, we subjected this gene set to enrichment analysis. A large number of significantly enriched annotations were observed in the analysis of GO terms (154 terms with q < 0.01) and KEGG pathways (115 pathways with q < 0.01) (S3 and S4 Tables). The top 4 GO terms (q < 7.6e^-18^) included positive and negative regulation of transcription from RNA polymerase II promoters (GO:0045944, GO:0000122), indicating a large fraction of transcription regulatory genes in this set. The term “anti-apoptosis” was listed on the 7^th^ position (q < 7.5e^-13^) with 64 annotations found, where only 16 are expected by chance. The top-three enriched KEGG annotations were “Pathways in cancer” (q < 1.6e^-92^), “Cytokine-cytokine receptor interaction” (q < 3e^-34^), and “Toll-like receptor signalling pathway” (q < 2.6e^-35^), with a range of cancer-related pathways following.

**Fig 6:**
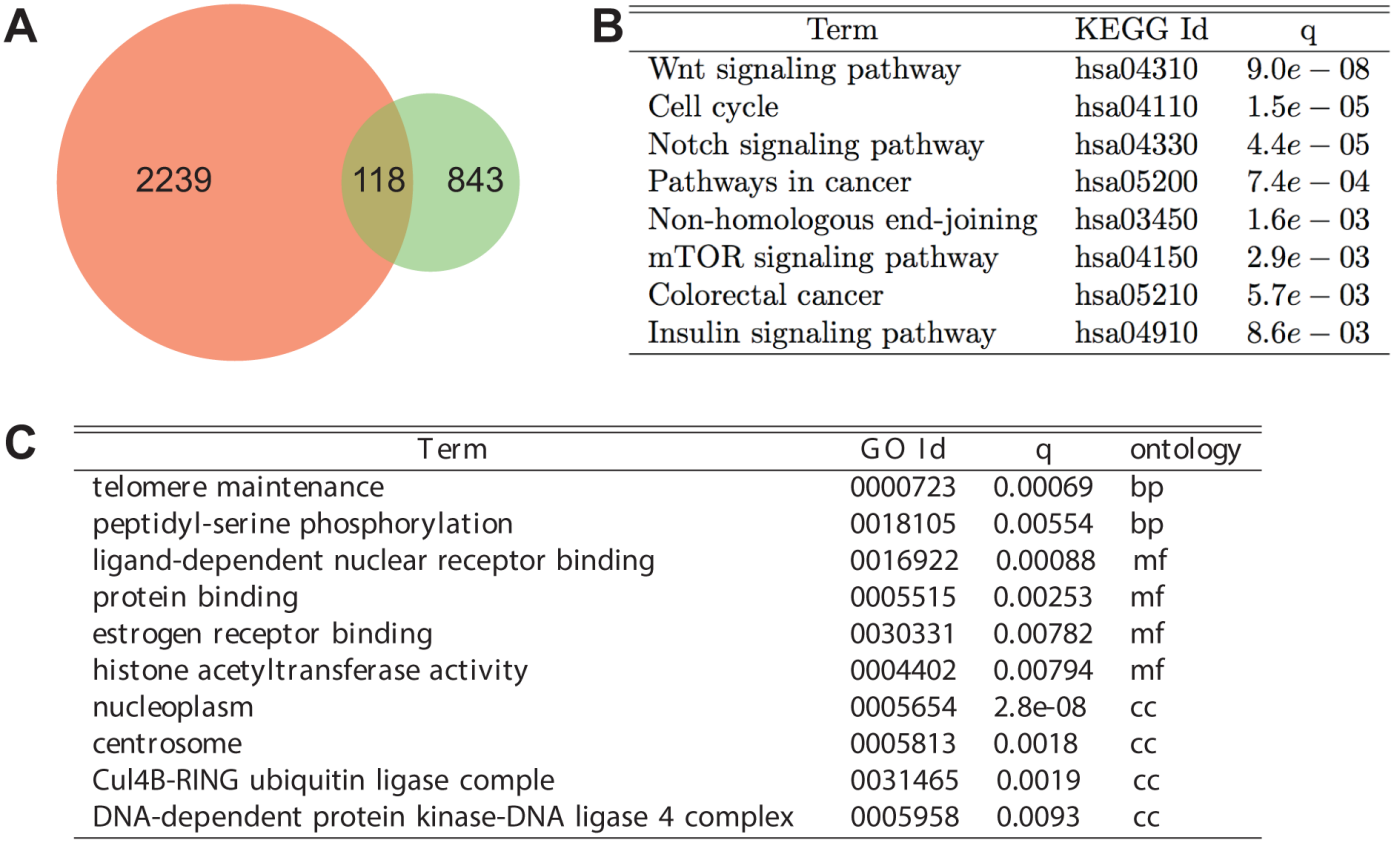
Functional analysis of the consensus predicted ECCN target gene set. Overlap between 2357 new ECCN elements (orange) based on expression pattern correlation and 961 genes obtained with text-mining methods (green) **(A)**. This resulted in 118 new genes found by both methods. KEGG pathway annotation enrichment of the 118 consensus predicted genes **(B)** and corresponding GO enrichment **(C).**

Intersecting the ECCN-interacting gene sets predicted by expression correlation (n = 2357) and text-mining (n = 961), respectively, resulted in a set of 118 genes (Fig 6A). While 38 novel interactions with an ECCN gene were predicted by both methods, 364 interactions were co-expression-specific and 182 were text-mining-specific (S5 Table). Interestingly, enrichment analysis of the 118 target genes using KEGG annotations indicated a strong connection to signalling- and cancer-related pathways (Fig 6B). The GO enrichment yielded the terms “telomere maintenance” and “peptidyl-serine phosphorylation” as significantly enriched biological processes (Fig 6C). The molecular function “ligand-dependent nuclear receptor binding” was also found to be significantly enriched (q < 0.0009).

**Fig 7:**
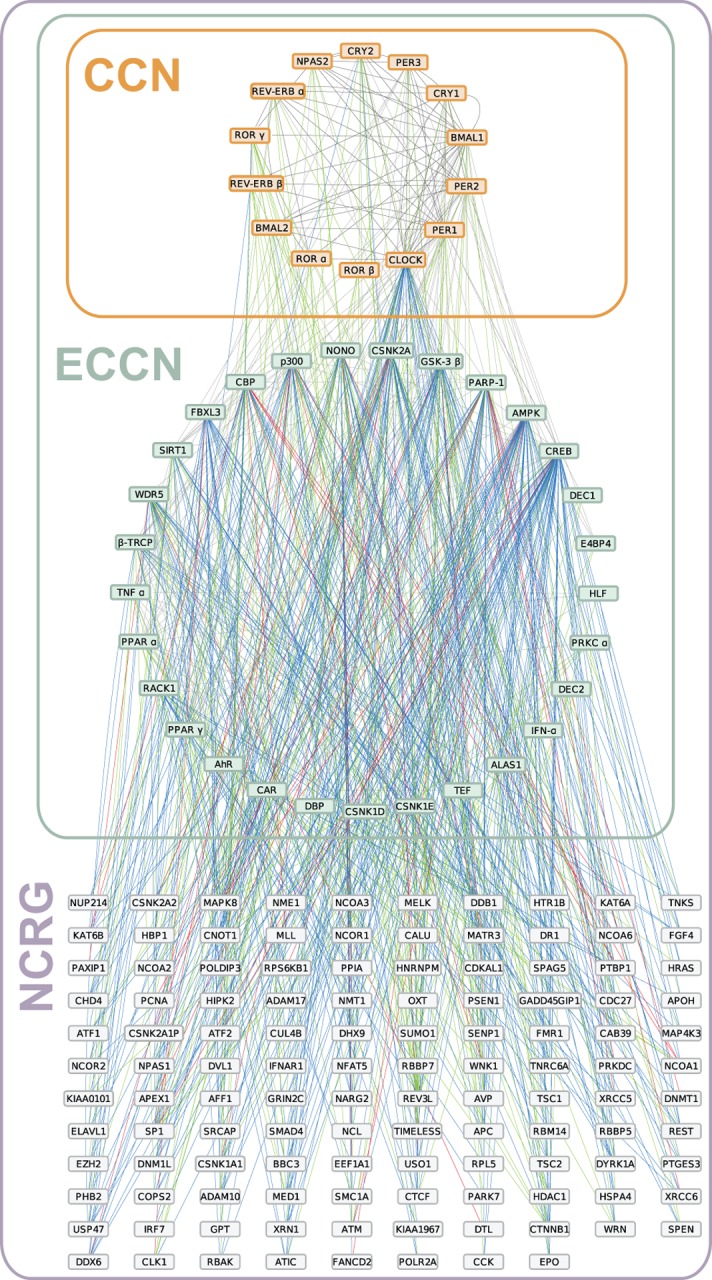
Network representation of CCN/ECCN network together with the 118 predicted target genes (NCRG). Boxes represent individual genes, which are connected by lines reflecting interactions that are known (grey), predicted by co-expression (blue), text-mining (green), or by both (red). The sub-networks are indicated by rectangles, the CCN (orange), the ECCN (green), and NCRG (purple).

Finally, we used this intersection of the text-mining analysis and co-expression analysis to extend the ECCN, resulting in a novel network of circadian regulated genes (NCRG) comprising 161 genes all together (Fig 7). An additional 220 interactions between the ECCN and the new NCRG were found amongst the text-mining dataset and 402 interactions within the co-expression data. The number of correlation-based interactions is less informative because, as we have shown above it is not a precise method to infer network topology. Since this assessment was derived from a mixture of various tissue types, the NCRG can be expected to be an aggregation of different tissue-specific interactions.

### Circadian phenotype amongst predicted ECCN extension genes

We tested how many of the 118 novel ECCN targets were found to exhibit circadian expression patterns in circadian data sets [14, 27]. Integration of these two mouse datasets, and mapping to human genes via HomoloGene yielded a total of 1771 circadian transcripts. These included the following 19 out of our 118 predicted targets (for p < 0.009, for p < 0.05 we find 59% genes out of the 118-set to be circadian): *Adam17*, *Apoh*, *Avp*, *Chd4*, *Clk1*, *Cops2*, *Ddx6*, *Dhx9*, *Dnm1l*, *Hnrnpm*, *Ifnar1*, *Map4k3*, *Ncl*, *Nmt1*, *Ncoa1*,*Psen1*, *Phb2*, *Smad4*, *Sumo1* (Table 3).

**Table 3:** Properties of the consensus predicted ECCN target genes.

		Chip-Seq Target Genes						Circadian Phenotype				Pathological phenotype
entrezID	Gene Symbol	RevErbα [16, 17]	RevErbβ [16, 17]	RevErbα/β [17]	RORα [22]	RORγ [22]	RORα/γ [22]	circadian expression	high-A	long-T	short-T	OMIM
102	ADAM10		x									Reticulate acropigmentation of Kitamura, 615537 (3)~{Alzheimer disease 18, susceptibility to}, 615590 (3)
328	APEX1	x										
350	APOH	x	x	x	x	x	x	x				
466	ATF1			x								
471	**ATIC**	x										AICA-ribosiduria due to ATIC deficiency, 608688 (3)
551	AVP							x				Diabetes insipidus, neurohypophyseal, 125700 (3)
813	CALU		x		x							
885	CCK	x										
996	CDC27			x								
1108	CHD4			x				x				
1195	CLK1							x				3MC syndrome 2, 265050 (3)
1386	ATF2											
1452	CSNK1A1	x	x	x						x		
1459	CSNK2A2			x								
1499	CTNNB1	x		x								Colorectal cancer, somatic, 114500 (3)~Hepatocellular carcinoma, somatic, 114550 (3)~Mental retardation, autosomal dominant 19, 615075 (3)~Ovarian cancer, somatic, 167000 (3)~Pilomatricoma, somatic, 132600 (3)
1642	**DDB1**			x					x			
1656	DDX6			x				x				
1660	DHX9							x				
1855	DVL1			x								
1859	DYRK1A	x	x	x								Mental retardation, autosomal dominant 7, 614104 (3)
1915	EEF1A1	x	x		x							
1994	ELAVL1											
2177	FANCD2			x								Fanconi anemia, complementation group D2, 227646 (3)
2547	XRCC6	x										
2875	GPT	x	x	x								
2905	GRIN2C	x	x	x								
3308	HSPA4					x						
3454	IFNAR1		x	x		x		x				
4089	SMAD4	x						x				Juvenile polyposis/hereditary hemorrhagic telangiectasia syndrome, 175050 (3)~Myhre syndrome, 139210 (3)~Pancreatic cancer, somatic, 260350 (3)~Polyposis, juvenile intestinal, 174900 (3)
4297	MLL	x	x	x								Leukemia, myeloid/lymphoid or mixed-lineage (2)~Wiedemann-Steiner syndrome, 605130 (3)
4299	AFF1	x	x	x								
4670	HNRNPM							x				
4691	NCL				x			x		x		
4830	NME1	x		x								Neuroblastoma, 256700 (3)
4836	NMT1	x	x					x				
5430	POLR2A				x							
5469	MED1			x		x						
5478	PPIA			x								
5599	MAPK8	x		x						x		
5663	PSEN1				x			x				Acne inversa, familial, 3, 613737 (3)~Alzheimer disease, type 3, 607822 (3)~Alzheimer disease, type 3, with spastic paraparesis and apraxia, 607822 (3)~Alzheimer disease, type 3, with spastic paraparesis and unusual plaques, 607822 (3)~Cardiomyopathy, dilated, 1U, 613694 (3)~Dementia, frontotemporal, 600274 (3)~Pick disease, 172700 (3)
5725	PTBP1	x		x								
5931	RBBP7											
5980	REV3L			x								
6125	RPL5			x								Diamond-Blackfan anemia 6, 612561 (3)
6667	SP1	x		x		x						
6868	ADAM17							x				Inflammatory skin and bowel disease, neonatal, 614328 (3)
7248	TSC1	x		x								Focal cortical dysplasia, Taylor balloon cell type, 607341 (3)~Lymphangioleiomyomatosis, 606690 (3)~Tuberous sclerosis-1, 191100 (3)
7341	SUMO1							x				Orofacial cleft 10, 613705 (3)
7520	XRCC5		x									
7994	KAT6A	x		x								
8021	NUP214			x								Leukemia, T-cell acute lymphoblastic (3)~Leukemia, acute myeloid, 601626 (3)
8202	NCOA3	x	x	x								
8491	MAP4K3	x	x					x				
8615	USO1	x	x	x								
8648	NCOA1							x				
9318	COPS2										x	
9611	NCOR1	x	x	x								
9612	NCOR2	x	x	x								
10059	DNM1L							x				Encephalopahty, lethal, due to defective mitochondrial peroxisomal fission, 614388 (3)
10432	RBM14		x	x	x	x	x					
10499	NCOA2	x										
10615	SPAG5		x		x	x	x					
10664	CTCF			x								Mental retardation, autosomal dominant 21, 615502 (3)
10725	NFAT5			x								
10728	PTGES3			x				x				
11331	PHB2							x				
23013	SPEN	x		x								Megakaryoblastic leukemia, acute (2)
26959	HBP1	x										
27113	BBC3	x		x								
27327	TNRC6A		x									
28996	HIPK2	x	x									
51514	DTL	x	x	x								
54464	XRN1			x								
55031	USP47		x	x								
57786	RBAK				x							
79664	NARG2	x	x	x	x							
90480	GADD45GIP1			x								

Genes marked in bold were found to be BMAL1 targets.

All 62 genes (of 118) are shown which exhibit at least one of the following properties: regulated by REV-ERB or ROR, circadian expression pattern, causing a clock phenotype upon RNAi knockdown, predicted as similar to known clock genes [29], and featuring an OMIM annotation.

Additionally, we were interested in the possible consequences of perturbing the newly identified genes in the circadian phenotype and checked whether any of the 118 predicted ECCN-interacting genes were found to cause perturbations on the circadian clock in available siRNA datasets [28, 29]. We found hits for different circadian phenotypes a) long-period phenotype: *Csnk1a1* (*casein kinase 1*, *A 1*), *Mapk8* (*mitogen-activated protein kinase 8*), *Ncl* (*nucleolin*); b) high-amplitude phenotype: *Ddb1* (*damage-specific DNA binding protein 1*); and c) short-period phenotype: *Cops2* (*COP9 signalosome subunit 2*). Among these, *Ncl* and *Cops2* also showed a circadian expression pattern. *Ncl* yielded a JTK q-value of 6.16e-06, a period of 24h, and a phase of 18.5. *Cops2* yields a p-value of 0.007, a period of 28h, and phase 2.5. These findings are summarized in Table 3.

We further compared our findings with a recent list of 1000 genes classified as—“sufficiently similar”—to known clock genes by a machine learning approach on a combination genome-scale datasets from mouse fibroblast cell lines [29]. One quarter of these genes were also contained in at least one of our gene sets (253 of 993 with a homolog in the human genome), and 10 genes were also detected by our text-mining and co-expression analysis: *Atf2*, *Ddx6*, *Dhx9*, *Elavl1*, *Hspa4*, *Ncl*, *Nme1*, *Med1*, *Rbbp7*, *Dnm1l*. Out of these, the four genes *Ddx6*, *Dhx9*, *Ncl*, and *Dnm1l* exhibit a circadian expression pattern.

### Clock target genes could be validated with ChIP-seq data

To further validate our 118 consensus genes gained from the bioinformatics approach, we examined the publicly available ChIP-seq datasets for REV-ERBα/β [16, 17]. Additionally, a BMAL1 dataset [12] was considered. ChIP-seq peak locations were used to calculate an association score (“ClosestGene” [30]) for each gene to the corresponding transcription factor. Simple threshold calculation then yielded a TF-target prediction. The gene association score S_g,tf_ was calculated for all annotated refSeq genes of the mouse genome build used in the corresponding experiment. The resulting log2 transformed S_g,tf_ distributions are shown in S7 Fig. The threshold for accepting a TF—gene association was chosen as 3, which yields the higher second gene-score peak in case of the bimodal REV-ERBβ peak set, or the prominent right shoulder of the distribution for all other peak sets (S7 Fig). A total of 3847 predicted REV-ERBα and 3388 REV-ERBβ target genes [16] were found. The alternative dataset provided 4618 target genes associated with REV-ERBα/β unspecific peaks [17]. Lastly, this procedure yielded 223 significant BMAL1 target genes [12]. Since the ChIP-seq peak location data for RORα and γ, were not accessible, we relied on the list of predicted targets provided by the authors based on a less stringent target prediction method [22, 31].

**Fig 8:**
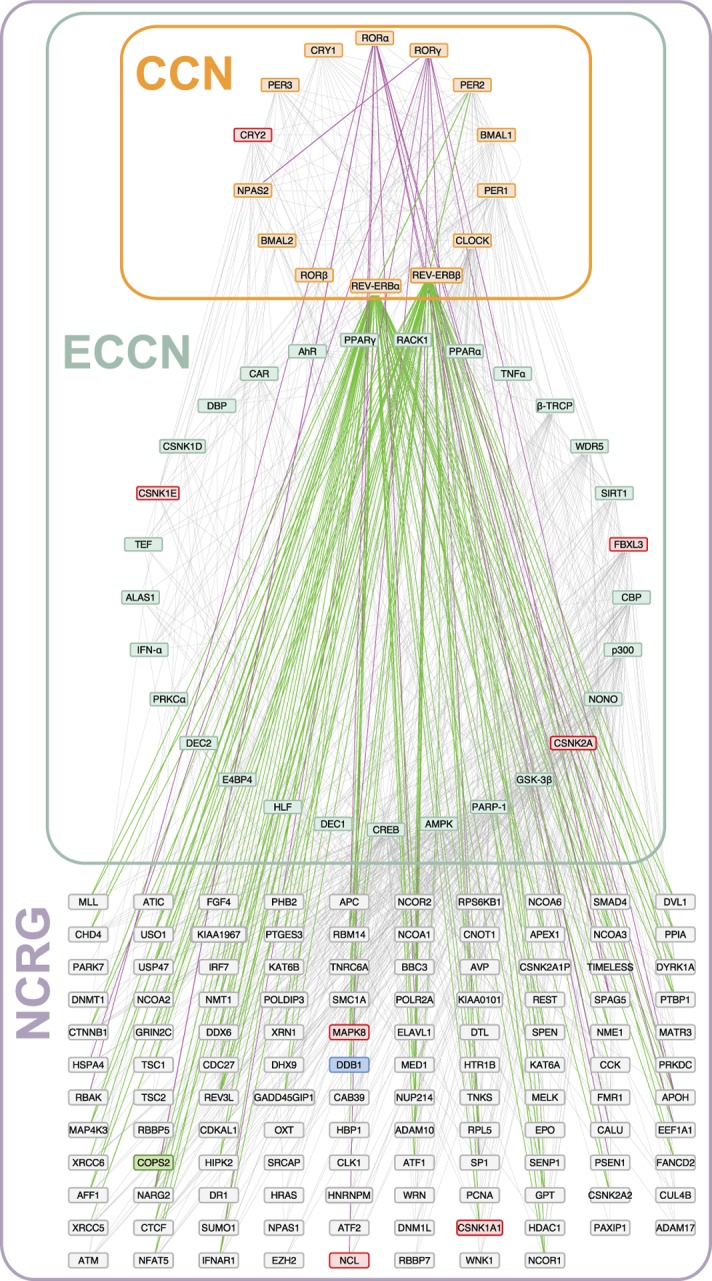
Transcriptional regulation of the CCN/ECCN extension network of 118 genes by REV-ERB and ROR. Regulatory interactions of REV-ERB α/β with NCRG genes (green lines) were derived from the locations of physical binding of these proteins in two ChIP-seq experiments [16, 17]. The ROR α/ γ interactions (purple lines) were adopted from the report of a third ChIP-seq experiment [22]. Genes with an observed phenotype in the genome-wide RNAi screen [28, 29] are shown with a coloured box, red indicating long period, blue a high amplitude, and green a short period.

Overall, we obtained a set of 118 genes potentially regulated by the ECCN. Of those, 19 exhibited circadian expression patterns, 5 exhibited phenotypic changes in the clock when targeted with RNAi, 59 were targeted by REV-ERBα/β, and 14 were targeted by RORα or γ. Additionally, the two NCRG genes *Ddb1* and *Mapk8* were found to associate with BMAL1 binding sites. These findings are summarized in Table 3 and depicted in Fig 8, (see S7 Table for all annotations).

## Discussion

The mammalian circadian clock is an endogenous, time-generating system with the peculiarity of synchronizing and propagating time-cues to the entire organism. Its relevance in the time-dependent regulation of biological processes has been shown at the organismal and cellular levels. As such, it is of no surprise that malfunctions of the circadian system were found to be associated to pathological phenotypes including obesity, sleep disorders and increasing incidence of cancer. The prospect of using individual patient-timing, based on the internal circadian clock, for therapy optimization is being explored with promising results. For instance, advances in chronotherapy have proven to be efficient in reducing toxicity and increasing efficacy in some types of cancer, particularly colon cancer [32]. A more detailed knowledge of the circadian network including the pathways it regulates is of major importance for the analysis on how time effects may be propagated and to determine the time-dependent action of certain drugs.

In this work we set up to dissect such clock-regulated pathways and to analyse the extent of circadian regulation at the cellular level by expanding the core circadian network to its potential target genes. We used human high-throughput transcriptome-data sets associated to text-mining of biomedical literature, for *de novo* clock regulated gene discovery.

### A network of circadian regulation: combining independent evidences

Gene co-expression has previously been used to predict gene functions. Such works rely on the Pearson correlation coefficient and extensions of it and, although able to predict gene functions in mammals, are limited in terms of *de novo* network generation [24, 25]. We observed that reportedly interacting ECCN genes feature correlation values which are similar to non-reported. This is a limitation of co-expression methodologies and the problem of erroneous transitive links inferred by correlation analysis was described before [33]. Therefore, we used a hybrid-methodology where to the expression correlation data we associated the text mining as an independent source of knowledge, enabling us to find regulated genes and their connection to the ECCN with increased confidence (Fig 1). This allowed us to partially overcome the limitations of expression analysis in terms of network topology and to be able to generate a semi-regulatory network for the mammalian circadian clock. Still, we do not analyse tissue-specificity issues which go beyond the scope of this work. Nevertheless, the circadian clock has been reported, in mammals, to be present in all cells so that the core network is expected to be very similar [34]. The output genes in the large network might indeed show tissue-specific differences which will be very interesting to explore in future work.

### Biological significance and impact in tumourigenesis

The detailed analysis of the network generated by our pipeline (NCRG) strengthens previous findings which associate the circadian clock to regulation of several molecular processes such as mRNA processing, cell division, cell cycle progression and DNA repair [19, 21, 35–40]. Particular pathways, including RNA transport, splicing and several cancer related pathways were identified by our study as being significantly associated with the circadian clock, highlighting the important function of the circadian system in the regulation of cellular processes. By comparing the difference in overrepresented terms between genes tightly- and those loosely-associated to the ECCN, we found that cell cycle and translation related terms are highly significant in loosely associated genes in comparison to tightly associated genes. We also found that splicing remains a highly over-represented term regardless of tightness (Fig 4). Together with the enriched biological processes such as “DNA-dependent regulation of transcription” and “gene expression”, it became clear that the co-expression based predicted ECCN target gene set has a stout emphasis on cellular signalling, transcriptional regulation, and cancer (Fig 5). Furthermore, several members of the predicted set of ECCN target genes are associated with Mendelian diseases as listed in the Online Mendelian Inheritance in Men dataset (OMIM) (S6 Table). 30% of the correlation/text-mining consensus genes featured such an annotation (35 of 118), pointing to the role of the circadian clock in pathogenesis.

In particular, among our top candidate genes is a group of genes associated with tumourigenesis (see Table 3): *Elavl1* is known to be highly expressed in several cancers and potentiates a characteristic pro-inflammatory profile of some immunological and non-immunological diseases [41], *Nme1* is considered a tumour suppressor and its expression is reduced in metastatic cancers [42], *Dhx6* belongs to the DEAD box helicase superfamily and is involved in DNA repair, *Med1* regulates p53-dependent apoptosis [43] and *Rbbp7* interacts with the tumour-suppressor gene *Brca1* [44] and may have a role in the regulation of cell proliferation and differentiation.

Remarkably, we found a subset of nine genes (*Apoh*, *Ifnar1*, *Sp1*, *Narg2*, *CALU*, *EEF1A1*, *RBM14*, *Spag5*, *Med1*) which are targets of both REV-ERB and ROR according to Chip-Seq experiments. These two nuclear receptors are known to bind RORE elements within the promoter regions of target genes: while REV-ERB is an inhibitor, ROR acts as an activator. APOH (Apolipoprotein H) and IFNAR1 (Interferon Alpha, Beta, Omega Receptor) are involved in immune disorders [45, 46]. SP1 (Sp1 transcription factor) is also involved in immune response and in many other cellular processes, including cell differentiation, cell growth, apoptosis, response to DNA damage, and chromatin remodelling [19]. NARG2 (NMDA receptor regulated 2) is associated to breast cancer [47], and *Med1* regulates p53-dependent apoptosis [43] and was found to be mutated in human carcinomas with microsatellite instability [48]. The eukaryotic translation elongation factor EEF1A1 was recently shown to mediate the alternative caspase-independent cell death mechanism induced by genetically unstable tetrapolidy [49]. The sperm associated antigen 5 (SPAG5) was found to be associated with various types of cancer, such as cervical cancer and breast cancer [50]. Circadian regulation of these genes and as such of the processes they regulate could be achieved via a fine-tuning of ROR/REV-ERB.

Two other circadian regulated genes identified by our study are *nucleolin* (*Ncl*) and *Ddx6*. The analysis of ChIP-seq data identified these genes as targets of RORγ and REV-ERBα, β, respectively. Interestingly, they were also reported to be involved in miRNA regulation [51–53]. DDX6 (RNA helicase) is found in p-bodies for mRNA degradation, needed for miRNA-mediated silencing. NCL regulates several miRNAs including miR-21, miR-221, miR222 and miR-103. miR-21 is defined as an oncogene and found to be overexpressed in most tumour types [51, 54–59], whereas miR-221 and miR222 show an increased expression in human breast cancer [60, 61]. Also, miR-222 was shown to promote resistance of cancer cells to cytotoxic T lymphocytes [62]. Interestingly, miR-103 which is also a target of NCL was reported to exhibit circadian pattern [63].

Altogether, our data allowed the generation of a large network of circadian regulation. The network was retrieved from human expression data intersected with text-mining of the biomedical literature, for topology refinement and *de novo* target identification. The novel predicted targets of the circadian clock network showed a remarkable association to cancer driving mechanisms. One of these mechanisms is miRNA regulation. Very recent studies point to an influence of miRNAs on the circadian clock [64–71], but only a few links on the regulation of miRNAs via the circadian clock have been described [69]. NCL represents a potential novel link via which the circadian clock, in particular RORγ, regulates the expression of miRNAs, with particular consequences in cancer progression.

## Methods

### Preprocessing

For all text-mining steps we used articles from PubMed and PubMed Central open access subset.

### Named entity recognition

Genes: For gene name recognition and normalization we used the GNAT library [72]. GNAT uses custom dictionaries and conditional random fields (CRF) for gene name recognition and subsequently normalises gene mentions to Entrez Gene ID’s. The system is ranked among the first in several critical evaluations [73, 74] and achieves, according to these assessments, a precision of 82% and recall of 82% for abstracts and 54/47% for full—text articles.

### Relation extraction

GeneView (a search engine which uses a comprehensively annotated database of all PubMed abstracts and 270,000 full texts from the open PubMed Central corpus) uses the shallow linguistic kernel [75] and LibSVM for relationship extraction between proteins. The model is trained on the ensemble of five publicly available training corpora [76]. This kernel achieved very good results in a comprehensive evaluation of nine machine learning kernels for PPI extraction from text [77–79]. Furthermore, is does not use dependency information and thus is very fast, a pre-requisite for usage in a large system such as GeneView. Data contained in GeneView is available at http://bc3.informatik.hu-berlin.de/. To account for species specificity, we mapped mammalian gene identifiers to Homologene clusters [80]. To test the efficiency of text-mining in contributing to new network generation, we first evaluated its ability to reconstruct a previously designed network of clock-controlled genes (CCGs) containing 121 interactions among 41 different proteins [19]. We used GeneView to extract all pairwise interactions. GeneView contained evidence for 73% of all interactions described in the network tested. The high sensitivity of the method encouraged us to further develop our pipeline in order to ascertain potential new elements and interactions. We further used GeneView to collect all interactions among the CCN and its directly interacting neighbours. After curation and filtering for direct interactions, we enriched the core-clock network with 108 novel interactions supported by 132 PubMed references, which led to the extended core-clock network (ECCN) recently reported [21]. For the ECCN, each candidate interaction is supported by up to 851 sentences (in total 4,206 sentences). We reduced the number of sentences to 580 by ranking them by confidence and returning only 5 sentences at maximum for each candidate. Sentences containing potentially novel PPI were ranked by the confidence of the classifier (ie. distance to the hyperplane) and were subsequently evaluated.

### Predicting interactions using coexpression data and overrepresentation of associated gene terms

Each dataset was assessed on the number of genes they share with the ECCN and how well the correlation coefficient distributions of known ECCN gene interactions were separated from a background distribution of all genes, where the Wilcoxon Rank Sum test was used for quantification. For more details on the dataset properties and selection, see S2 Text.

To find associated genes based on the correlation coefficients, we selected the 10000 highest correlations between any ECCN gene and a non-ECCN gene as predicted interactions, thereby considering the 1.18% most extreme correlation values.

We sought to detect and characterize only genes that were tightly associated with the ECCN, where "tightness" was defined as the number of connections between a gene and a set of genes. Accordingly, the comparison of the number of predicted NCRG with required tightness 1 to 10 shows the most drastic decline between 1 and 2, which quickly diminishes with rising tightness values (Fig 4). We therefore chose to employ a tightness threshold of 2 for the remaining analysis. We then proceeded to find the overrepresented terms and enriched clusters using the R package TopGO [81]. We annotated the associated genes with terms from the Genetic Association Database[82], Online Mendelian Inheritance in Man database[83], Swissprot Protein Information Resource [84], Gene Ontology [85], Pubmed and Kyoto Encyclopedia of Genes and Genomes [86]. Significant overrepresentation was determined using p-values corrected by Benjamini-Hochberg multiple testing correction (q-values).

### Integration of the predicted NCRG with transcriptional features

We compared our NCRG prediction with the machine learning based prediction of clock genes [29]. Therefore, we retrieved the top 1000 genes as of the evidence factor ranks and used the HomoloGene database build 66 [80] to map the reported mouse genes to 993 unique entrez genes, could then be compared to our predicted genes set.

Similarly, we tested how many of the NCRG are amongst the genes with circadian expression regulation according to recent publications [14, 27]. After combination of both lists of mouse genes, a total of 1771 unique entrez transcripts were obtained for comparison after mapping via HomoloGene build 68.

An extensive collection of genes which lead to circadian clock phenotypes upon knockout via RNAi has been described recently [28]. The reported 343 genes are categorized into double hitters, i.e. two different pairs of siRNAs lead to a circadian clock phenotype, and single hitters, for which only one of the two siRNA pairs designed for each gene lead to a phenotype, where amplitude- and phase-changes were considered as phenotype.

### ChIP-seq data analysis

We employed the R package TFTargetCaller [30] to derive target gene sets for clock-related transcription factors from experimental Chip-seq data using the method “ClosestGene”. We used available data sets to extract target genes for REV-ERB α/β [16, 17] and for BMAL1 [12]. These include all available Chip-seq data sets for core-clock genes. Specifically, the genomic peak locations were obtained, and the gene association score S_g,tf_ was calculated for all annotated refSeq genes of the mouse genome build used in the corresponding experiment. The resulting log2 transformed S_g,tf_ distributions are shown in S7 Fig. The threshold for accepting a TF—gene association was chosen as 3, which yields the higher second gene-score peak in case of the bimodal REV-ERB β peak set (S7B Fig), or the prominent right shoulder of the distribution for all other peak sets. Since the genomic locations of the peaks for the ROR α/γ dataset were not available, we used the predicted target list provided by the authors [22, 31].

## Supporting Information

S1 Fig Correlation distributions for clock network gene pairs versus random gene pairs. Cumulative correlation value distributions obtained from the HSA dataset, shown for comparison with Fig 3 in the main text.
(EPS)

S2 Fig Correlation of reported CCN interactions, ECCN interactions as compared to random background. The Pearson ρ distributions of pairs of ECCN genes reported to interact but excluding CCN (ECCN, green), reported pairs of CCN genes (CCN, orange), and 43 randomly chosen genes versus all genes as background (BG, black) are shown. Pearson correlation coefficient **(A, C)** and mutual rank **(B, D)** probability density functions for known CCN interactions and reported ECCN interactions compared with random background. Data extracted from the HSA and HSA2 dataset are shown in **(A, B),** and **(C, D)** respectively. Shown for comparison with Fig 3 in the main text.
(EPS)

S3 Fig Correlation of reported ECCN interactions compared to not-reported interactions. Pearson correlation coefficient **(A, C)** and mutual rank **(B, D)** probability density functions for all ECCN interactions (i.e. including all CCN interactions, “reported ECCN”) compared with all other possible pairs between ECCN genes, for which no interaction is reported (“other”). Data extracted from the Hsa and Hsa2 dataset are shown in **(A, B),** and **(C, D)**, respectively. Shown for comparison with Fig 3 in the main text.
(EPS)

S4 Fig Correlation of the ECCN interactions and the background. Pearson correlation coefficient **(A, C)** and mutual rank **(B, D)** probability density functions for all possible pairs of ECCN genes compared with all other possible pairs between one of the 43 ECCN genes and a non-ECCN gene as background. Data extracted from the Hsa and Hsa2 dataset are shown in **(A, B),** and **(C, D)**, respectively. Shown for comparison with Fig 3 in the main text.
(EPS)

S5 Fig Tightness filtering effect. The number of predicted target genes (y-axis) decreases when increasing the minimal number of ECCN genes, with which it has to correlate (x-axis).
(EPS)

S6 Fig Number of interactions (x-axis) for 32 ECCN genes (y-axis) as predicted by expression correlation. The remaining 11 ECCN genes do not feature predicted targets.
(EPS)

S7 Fig Association scores for core clock transcription factors. Association strength scores S_tf,g_ between the core clock transcription factors REV-ERB α/β and BMAL1 and all refSeq genes annotated in the corresponding genome version (mm8 [17] or mm9 otherwise) were calculated using the “ClosestGene” method of the R package TFTargetCaller and the ChIP-seq peak annotations. The number of genes with S_tf,g_ > 0 is shown as n_gene_, the cutoff for accepted TF-gene association was set to 3 as marked with a red dashed line, and the number of accepted target genes is shown as n_target_ for each individual dataset.
(EPS)

S8 Fig Cytoscape file for Fig 7. (http://www.cytoscape.org/).
(ZIP)

S9 Fig Cytoscape file for Fig 8. (http://www.cytoscape.org/).
(ZIP)

S1 Table List of ECCN interactions with publication references obtained by text-mining. (XLS)

S2 Table Extension of the ECCN network using text-mining method. (XLSX)

S3 Table List of GO term annotations enriched amongst the ECCN target genes predicted by text-mining. The table provides the term ids (“GO.ID”), the corresponding “Term”, as well as the overrepresentation p-value after false discovery rate correction after Benjamini-Hochberg (“pval”). In addition, the total number of genes annotated with the respective term is provided (“Annotated”), the number of significant annotations (“Significant”), along with the number of annotations expected by chance in the gene set (“Expected”).
(XLSX)

S4 Table List of KEGG pathway annotations enriched amongst the ECCN target genes predicted by text-mining. The table provides the pathway ids (“kegg.id”), the corresponding pathway “name”, as well as the overrepresentation p-value before (“pval”) and after false discovery rate correction after Benjamini-Hochberg (“fdr”).
(XLSX)

S5 Table List of all newly predicted interactions. The columns “gene1.entrez”, “gene2.entrez”, “gene1.symbol”, and “gene2.symbol” provide the Entrez ids and gene symbols for the two predicted interacting genes, respectively. The Boolean flags “txtmn” and “coxp” indicate interactions predicted by text-mining, and co-expression, respectively. “with.consensus” indicates interactions involving one of the 118 consensus genes, and “overlapping” indicates the interactions predicted similarly by text-mining and co-expression.
(XLSX)

S6 Table Characterization of the 118 predicted NCRG regarding disease-related annotation. Annotations from OMIM, gad, and the KEGG database were integrated in addition to SNPs. Genetic association database (gad), KEGG pathway annotation, traits that significantly associate with the gene as provided in the Ensemble database (gwas_trait, gwas_pval, gwas_pubmed_id), the number of non-synonymous SNPs found in the gene (nonsyn_count, nonsyn_norm), the Uniprot database derived protein domains (up_seq_feature), the Gene Ontology biological processes annotations (goterm_bp), and the Gene Reference into Function (generif).
(XLS)

S7 Table Characterization of the 118 NCRG regarding expression regulation by transcription factors. The transcription factor target prediction of all 118 NCRG for both REV-ERBα/β datasets, the BMAL1, and also the RORα/γ dataset is provided. Additionally, the phenotype upon gene knockdown observed [28] and the prediction of “similar to clock gene” [29] are included. The observed circadian expression and OMIM annotations are indicated.
(XLS)

S1 Text Text-mining-based assembly and characterization of the ECCN network. (DOCX)

S2 Text Comparison of available co-expression databases. (DOCX)
