# Supplementary material for: Assembly of a Comprehensive Regulatory Network for the Mammalian Circadian Clock: A Bioinformatics Approach
Source: PLoS One. 2015 May 6;10(5):e0126283. doi: 10.1371/journal.pone.0126283 (PMC4422523; doi:10.1371/journal.pone.0126283)
Supplement: S1 Text — (DOCX) [file pone.0126283.s017.docx]

**Text S1 – Text mining-based assembly and characterization of the ECCN network**

To further characterize the new ECCN, we used our text-mining tools to annotate particular key-entities related to the ECCN genes.

Our text mining software – GeneView (see Materials and Methods) a best-of-breed strategy, annotations in are of high quality, with estimated precision and recall quantification termed F-measure [1] ranging (depending on the entity class) between 96% for species and 69% for chemical names. We annotated all genes, histone modifications, species, chemicals, single nucleotide polymorphisms (SNPs), drugs, tissues, diseases, circadian clock and chronotherapy, using high-quality named entity recognition tools. Subsequently the GeneView repository contains more than 192 k entities for 10 different entity types. Protein-protein interactions are identified using the shallow linguistic kernel. This step results in the annotation of almost 3,9 million PPIs in about 800,000 articles. We evaluated the performance of our software in finding supporting sentences for pairwise interactions (Figure 1) for all pathways contained in Kegg and Reactome. The results in Table 1 show species specific results for pathway reconstruction. The exact distribution of all found entities is shown in Table 2 and illustrated in Figure 2. The number of different entities per type ranges from 132 to 110,880. Overall, 13,522,014 articles are annotated with at least one entity. The most frequent entity type is chemical compound, with about 47% of all articles containing at least one chemical entity. Species, drug, and gene names also occur comparably frequent. histone modifications, species, chemicals, single nucleotide polymorphisms (SNPs), drugs, tissues, diseases, circadian clock and chronotherapy. Interestingly, all named entities not-related to the circadian clock key-terms showed a strong increase in the amount of published manuscripts, in the last 24 years. In contrast, for circadian terms associated to ECCN components, the increase in the number of publications was much scarcer and it took place within the last 10 years, which relates to the newness of the field. Even more striking is the association of ECCN entities with the term *chronotherapy*, which resulted in 24 published reports in PubMed, within the last 24 years. Yet recent advances in chronotherapy have proven to be efficient in reducing toxicity and increasing efficacy in some types of cancer, particularly colon cancer [2]. This highlights the importance of better describing the circadian system and stands as a strong motivation for further discovery of a circadian gene regulatory network.

**A**

**B**

**Figure 1.** Reconstruction performance by the minimal number of supporting sentences for pathways contained in Kegg and Reactome. **(A)** Performance depending on the minimal number of co-occurring proteins. **(B)** Performance depending on the minimal number positively classified protein pairs.

| **Performance** | | | | | |
| --- | --- | --- | --- | --- | --- |
| **Species** | **Precision** | **Recall** | **F1** | **Pathways** | **Interactions** |
| **Human** | 45.8 | 14.5 | 22.0 | 527 | 27,132 |
| **Mouse** | 40.9 | 8.4 | 13.9 | 436 | 12,866 |
| **Rat** | 41.1 | 12.1 | 18.6 | 337 | 2,729 |
| **Fly** | 78.9 | 5.1 | 9.6 | 168 | 4,605 |
| **Arabidopsis** | 100.0 | 5.1 | 9.6 | 37 | 419 |

**Table 1.** Reconstruction performance for different species ordered by the number of species-specific interactions.

| **Entitiy** | **Articles** | **Entities** | **Unique** |
| --- | --- | --- | --- |
| **Cell-type** | 36,851 | 82,285 | 585 |
| **Chemicals** | 9,851,347 | 73,354,240 | 59,232 |
| **Disease** | 272,240 | 679,364 | 9,681 |
| **Drugs** | 6,023,081 | 44,595,216 | 3,052 |
| **Genes** | 2,855,898 | 31,383,852 | 81,229 |
| **Histones Mod.** | 5,938 | 62,370 | 316 |
| **SNP** | 163,938 | 815,239 | 14,302 |
| **Species** | 8,815,334 | 40,992,161 | 110,880 |
| **Tissue** | 8,164 | 9,488 | 132 |

**Table 2.** Overview of all entities found and contained in the GeneView repository. Articles: number of citations with at least one entity found. Entities: total number of recognized mentions. Unique entities: number of distinct entities.

**Figure 2.** Number of citations tagged with at least one specific entry type.

As a comparison we performed a full pub med search for all ECCN elements (Figure3)

| **Entitiy** | **Articles** | **Normalized** | **Unique** |
| --- | --- | --- | --- |
| Cell-type | 1,065 | 2,804 | 260 |
| Chemicals | 64,018 | 971,015 | 11,562 |
| Disease | 59,321 | 721,194 | 7,040 |
| Drugs | 48,865 | 666,123 | 1,783 |
| Genes | 73,186 | 2,589,230 | 24,956 |
| Histones | 1,045 | 15,468 | 238 |
| Mutations | 4,840 | 25,717 | 5,207 |
| Species | 47,704 | 683,144 | 5,240 |
| Tissue | 157 | 188 | 41 |
| Circadian KeyTerms | 1,474 | — | — |
| chrono KeyTerms | 24 | — | — |
|  |  |  |  |

**A**

**B C**

**Figure 3.** Number of citations tagged with at least one specific entity type.

**(A)** Characterization of the ECCN, in terms of citations related to: histone modifications, species, chemicals, single nucleotide polymorphisms (SNPs), drugs, tissues and diseases. **(B)** Frequency of terms, related and not-related to the circadian clock, in the literature (associated to our genes of interest) in the last 24 years. **(C)** Frequency of terms *circadian clock* and *chronotherapy* in the literature (associated to our genes of interest) in the last 24 years.

References

1. Manning C., S.H., *Foundations of Statistical Natural Language Processing*. 1999, Cambridge, MA: MIT Press.

2. Lévi, F., et al., *Implications of circadian clocks for the rhythmic delivery of cancer therapeutics.* Advanced drug delivery reviews, 2007. **59**: p. 1015-35.

1. Lévi, F., et al., *Implications of circadian clocks for the rhythmic delivery of cancer therapeutics.* Advanced drug delivery reviews, 2007. **59**: p. 1015-35.

2. Manning C., S.H., *Foundations of Statistical Natural Language Processing*. 1999, Cambridge, MA: MIT Press.

1. Lévi, F., et al., *Implications of circadian clocks for the rhythmic delivery of cancer therapeutics.* Advanced drug delivery reviews, 2007. **59**: p. 1015-35.

2. Manning C., S.H., *Foundations of Statistical Natural Language Processing*. 1999, Cambridge, MA: MIT Press.

1. Manning C., S.H., *Foundations of Statistical Natural Language Processing*. 1999, Cambridge, MA: MIT Press.
