# Supplementary material for: Assembly of a Comprehensive Regulatory Network for the Mammalian Circadian Clock: A Bioinformatics Approach
Source: PLoS One. 2015 May 6;10(5):e0126283. doi: 10.1371/journal.pone.0126283 (PMC4422523; doi:10.1371/journal.pone.0126283)
Supplement: S2 Text — (DOCX) [file pone.0126283.s018.docx]

**Text S2 – Expression-correlation data and the ECCN network**

To find a gene expression dataset that was suitable for building a co-expression network, we performed a literature search and collected all existing co-expression databases that could be used for our purposes. We examined two different published databases which provided a total of five data sets [1-3] for their ability to reproduce the ECCN.

Firstly, a collection of human B-cell microarray samples was presented by Nayak *et al*. [1]. The authors describe separation of these samples into subgroups termed asn, ceph-uath and yri. However, the pairwise Pearson correlation coefficients among all ECCN genes were poorly separated from the background of the correlation coefficients among all genes (p-values of 0.72, 0.09, and 0.41 by Wilcoxon Rank Sum test).

Secondly, the COXPRESdb database provides the pre-computed pairwise weighted Pearson correlation values between all genes probed by microarrays across a large number of samples from various experiments [2]. Two different human data sets are available, referred to as HSA and HSA2 and distinguished by the underlying microarray platform. Version 5.0 of the human correlation dataset HSA is based on 73083 samples of the microarray platform HG U133 Plus 2, introduced in 2003, covering 19803 genes. In contrast, HSA2 is based on 6865 samples of 19788 genes of the newer array design (HuGene-1 0-st-v1).

The following protocol was used for pre-processing the samples in each dataset. 1) The raw data were MAS5.0 summarized and log2 transformed. 2) Pairwise weighted Pearson correlation coefficients ρ were calculated between all possible probe combinations. 3) When multiple probes for a gene were available, the maximal correlation coefficient was used. Throughout this text, correlation values are referred to as ρ for HSA and ρ_HSA2_  for the HSA2 dataset. 4) Finally, the mutual rank (MR) measure was calculated from the Pearson ρ, following the reported procedure [4].

While correlations from the two datasets correspond as expected (Figure S1 left), systematic differences were observed. The HSA2 dataset features favourable statistical properties of the entire dataset, firstly it is less skewed, and secondly the standard deviation σ is larger (Figure S1 right). Furthermore, only the ρ_HSA2_ distribution was centred on 0 (1^st^ quantile -0.076, median 0.0008, mean 0.01, 3^rd^ quantile 0.089, σ = 0.14), whereas the ρ_HSA_ dataset was skewed towards positive correlation values (1^st^ quantile -0.01, median 0.037, mean 0.054, 3^rd^ quantile 0.1, σ 0.1, see Figure S1 right). As demonstrated in the main text, the detected correlation signal of the CCN/ECCN gene set is also consistently larger in the HSA2 dataset.

**Figure S1.** Comparison of weighted Pearson ρ values derived from COXPRES collections Hsa2 on the x-axis versus Hsa on the y-axis **(A).** Distribution of COXPRES weighted Pearson ρ values from the Hsa and Hsa2 datasets **(B).**

References

1. Nayak, R.R., et al., *Coexpression network based on natural variation in human gene expression reveals gene interactions and functions.* Genome research, 2009. **19**(11): p. 1953-62.

2. Obayashi, T., et al., *COXPRESdb: a database of coexpressed gene networks in mammals.* Nucleic acids research, 2008. **36**(Database issue): p. D77-82.

3. Prieto, C., et al., *Human gene coexpression landscape: confident network derived from tissue transcriptomic profiles.* PloS one, 2008. **3**: p. e3911.

4. Obayashi, T. and K. Kinoshita, *Rank of correlation coefficient as a comparable measure for biological significance of gene coexpression.* DNA research : an international journal for rapid publication of reports on genes and genomes, 2009. **16**(5): p. 249-60.
